# *ELANE* rs17223045C/T and rs3761007G/A variants: Protective factors against COVID-19

**DOI:** 10.17305/bb.2023.9940

**Published:** 2024-06-01

**Authors:** José Manuel Fragoso, Gilberto Vargas-Alarcón, Ángel Emmanuel Martínez-Flores, Isela Montufar-Robles, Rosa Elda Barbosa-Cobos, Gustavo Rojas-Velasco, Julian Ramírez-Bello

**Affiliations:** 1 Laboratorio de Biología Molecular, Instituto Nacional de Cardiología Ignacio Chávez, México City, México; 2 Dirección de Investigación, Instituto Nacional de Cardiología Ignacio Chávez, México City, México; 3 Unidad de Investigación, Hospital Juárez de México, México City, México; 4 Servicio de Reumatología, Hospital Juárez de México, México City, México; 5 Unidad de Cuidados Intensivos, Instituto Nacional de Cardiología Ignacio Chávez, México City, México; 6 Subdirección de Investigación Clínica, Instituto Nacional de Cardiología Ignacio Chávez, Mexico City, Mexico

**Keywords:** Severe acute respiratory syndrome coronavirus 2019 (SARS-CoV-2), coronavirus disease 2019 (COVID-19), ELANE, single nucleotide variants (SNVs)

## Abstract

Severe acute respiratory syndrome coronavirus 2 (SARS-CoV-2) is responsible for causing coronavirus disease 2019 (COVID-19). The development and severity of this infectious disease is influenced by a combination of environmental and genetic factors. Angiotensin-converting enzyme 2 (ACE2) facilitates SARS-CoV-2 entry into human cells, with transmembrane serine protease 2 (TMPRSS2) playing a crucial role in S protein priming. Other proteases, such as cathepsin L and elastase, neutrophil-expressed (ELANE), have the capability to prime the S protein and contribute to SARS-CoV-2 infection. *ELANE* variants have not been previously examined in COVID-19 patients. We aimed to assess the association of single nucleotide variants (SNVs) within *ELANE* with COVID-19 and biochemical markers. The study included 319 SARS-CoV-2-infected patients and 288 controls. Genotyping of *ELANE* rs17216663C/T (Pro257Leu), rs17223045C/T (As1n30Asn), and rs3761007G/A was conducted using a 5’-nuclease allelic discrimination assay (TaqMan assay). Our findings indicate that *ELANE* rs17223045C/T (C vs T: odds ratio [OR] 0.08, *P* ═ 0.005, and CC vs CT: OR 0.08, *P* ═ 0.005) and rs3761007G/A (G vs A: OR 0.38, *P* ═ 0.009, and GG vs GA: OR 0.40, *P* ═ 0.008) confer protection against COVID-19. However, these variants were not associated with biochemical markers. In conclusion, our data suggests that *ELANE* rs17223045C/T and rs3761007G/A SNVs may play a protective role against COVID-19.

## Introduction

A disease characterized by atypical pneumonia was initially reported in Wuhan, Hubei Province, China, in late 2019 [1]. It was subsequently revealed that this infectious disease was caused by a novel coronavirus, identified as severe acute respiratory syndrome coronavirus 2019 (SARS-CoV-2) [2]. On March 11, 2020, the World Health Organization (WHO) officially declared the coronavirus 2019 (COVID-19) outbreak a global pandemic [3]. SARS-CoV-2, the causative agent, is an enveloped, positive-sense, single-stranded RNA virus with a genome comprising nearly 30,000 nucleotides and is composed of various structural and non-structural proteins [4]. The spike (S) structural protein, crucial for SARS-CoV-2 entry into the host cell, engages with the human angiotensin-converting enzyme 2 (ACE2) receptor through the receptor binding domain (RBD) [4, 5]. The priming of the S protein involves the participation of serine proteases, including transmembrane protease serine 2 (TMPRSS2) [6]. Additionally, other proteases, such as furin, cathepsin L, and elastase, neutrophil-expressed (ELANE), play a role in the priming of the S protein [7–10]. ELANE is expressed and released by activated neutrophils, gathering at the site of pathogen invasion to elicit an immune response [10]. Increasing evidence indicates altered protease levels among COVID-19 patients, with recent reports indicating upregulation in nasopharyngeal swabs [11]. Another study revealed a significant increase in ELANE blood levels among patients compared to controls. Elevated ELANE levels were associated with intensive care admission, body temperature variations, lung damage, markers of cardiovascular outcomes, renal failure, etc. Importantly, ELANE emerged as an independent predictor of the computed tomography score for COVID-19-related lung damage [12]. It was also reported that serum levels of ELANE in COVID-19 patients were significantly higher than those in healthy subjects, and especially elevated in patients in intensive care units (ICU) compared to non-ICU patients. Furthermore, this suggests that ELANE serves as an indicator of COVID-19 activity [13]. Taken together, these findings strongly imply a crucial role for ELANE in the pathogenesis of COVID-19.

On the other hand, Vargas-Alarcón et al. reported various *ELANE* single nucleotide variants (SNVs) that could play a significant role in susceptibility to or protection against COVID-19. Moreover, these variants may potentially exert a functional effect on the activity of the ELANE protein or its mRNA levels [8]. Despite research efforts, to explore *ELANE* variants in patients with COVID-19, as of now, no study has yet examined the potential association between *ELANE* SNVs and this infectious viral disease.

Some authors have reported associations of different *ELANE* mutations or SNVs in diseases, such as severe congenital neutropenia [14], end-stage chronic kidney disease [15], lung cancer [16], and coronary heart disease [17], among others. However, the role of various *ELANE* SNVs in patients with COVID-19 remains unexplored. We, therefore, focused on three *ELANE* SNVs previously suggested by Vargas-Alarcón et al. as potential COVID-19 markers, considering their possible effects on the protein structure or function. Two are located in the coding sequence: one is a non-synonymous variant, rs17223045C/T (Pro257Leu), and the other is a synonymous variant, rs17216663C/T (Asn130Asn). The third, rs3761007G/A, resides in the promoter or near the 5’ region, with predictions suggesting an impact on the binding of the DR4 transcription factor and potentially influencing mRNA expression.

Therefore, the objective of our study was to assess the potential impact of these three *ELANE* SNVs on susceptibility to or protection against COVID-19.

## Materials and methods

### Characteristics of study population

Our study comprised 319 confirmed cases of SARS-CoV-2 infection (verified by RT-PCR test) and 288 controls from Mexico. Patient recruitment took place from April 2020 to February 2021 at the Hospital Juárez de México and the Instituto Nacional de Cardiología Ignacio Chávez. The COVID-19 diagnosis, with a mean age of 55.4 ± 14.2 years, relied on clinical characteristics, such as loss of taste and odor, dry cough, fatigue, fever, diarrhea, chills, nasal congestion, sore throat, conjunctivitis, headache, musculoskeletal pain, skin rashes, dizziness, heart rate, and oxygen saturation. A positive PCR test for SARS-CoV-2 further confirmed the diagnosis. The exclusion criteria for our patients were as follows: individuals under 18 years of age, those coinfected with other viruses, patients with non-Mexican ancestry, individuals who had received transfusions in the last two weeks, pregnant patients, and those who passed away before a blood sample could be collected. In contrast, the inclusion criteria encompassed individuals hospitalized with severe or critical COVID-19 (requiring ICU admission for high-flow oxygen therapy or mechanical ventilation) or those with asymptomatic/mild disease. The control group consisted of 288 healthy individuals (mean age of 33.1 ± 7.6 years) affiliated with the Instituto Nacional de Cardiología Ignacio Chávez, all of whom were actively involved in the ICU with COVID-19 patients. This control group comprised medical residents, laboratory personnel, and nurses. Controls underwent a SARS-CoV-2 antibody test (Elecsys^®^ Anti-SARS-CoV-2, Roche Diagnostics International Ltd. CH-6343, Rotkreuz, Switzerland) to ensure the absence of SARS-CoV-2 infection.

### Analysis of laboratory and biochemical markers

Blood samples from cases and controls were processed within a class II biological safety cabinet following the institutional security protocols and the guidelines established in the Official Mexican Standards NOR-007-SSA3-2011, NOM-087-SEMARNAT-SSA1-2002, NOM-010-SSA2-2010, and NMX-EC-15189 IMNC-2015. In COVID-19 patients, we assessed various laboratory parameters, including creatinine, ferritin, lactic acid dehydrogenase (LDH), C-reactive protein (CRP), total bilirubin, aspartate aminotransferase (AST), alanine aminotransferase (ALT), hemoglobin, and platelet count. Additionally, we evaluated clinical features, such as cough, dyspnea, chest pain, headache, etc., and comorbidities such as type 2 diabetes mellitus, obesity, and hypertension. Detailed information is provided in Table 1.

**Table 1:** Demographic characteristics, clinical signs, and symptoms in patients with COVID-19

**Characteristics**	**COVID-19 patients (*n* ═ 320)**
Age (years)	55.4 ± 14.2
Sex, *n* (%)	207 (65) Male
	113 (35) Female
**Clinical signs and symptoms**	***N* (%)**
Cough	204 (63.7)
Dyspnea	198 (61.8)
Mechanical ventilation	112 (35)
Myalgia	111 (34.6)
Fatigue	111 (34.6)
Fever	86 (26.8)
Headache	84 (26.2)
Odynophagia	56 (17.5)
Diarrhea	39 (12.1)
Chest pain	32 (10)
Rhinorrhea	19 (5.9)
Nausea	17 (5.3)
Emesis	17 (5.3)
Abdominal pain	17 (5.3)
Temperature ^∘^C	36.64 ± 1.04
Oxygen saturation (SpO2)	85.03 ± 12.27
Heart rate	89.51 ± 20.12
**Comorbidities**	***N* (%)**
Obesity	211 (65.9)
Hypertension	129 (40.3)
T2DM	109 (34.0)
**Biochemical markers**	**Concentration**
Ferritin (ng/µL)	679 (358–1114)
LDH (U/dL)	353 (258.8–469.3)
Platelets (10^9^/L)	259 (189–351)
ALT (U/dL)	40.8 (24.8–69)
AST (U/dL)	41.2 (27.4–65.2)
C reactive protein (mg/dL)	17.7 (6.08–94.6)
Hemoglobin (g/dL)	13.9 (11.4–15.3)
Creatinine (mg/dL)	0.90 (0.69–1.35)
Total bilirubin (mg/dL)	0.60 (0.43–0.87)

Data are expressed as mean ± standard deviation, percentage, and median percentiles (25^th^–75^th^). LDH: Lactic acid dehydrogenase; ALT: Alanine transaminase; AST: Aspartate aminotransferase; T2DM: Type 2 diabetes mellitus; COVID-19: Coronavirus disease 2019.

### DNA isolation and genetic analysis

DNA isolation from peripheral blood was conducted following the method described by Goud et al. [18]. In brief, a 5 mL peripheral blood sample was collected in a K2 EDTA-vacutainer tube. After centrifugation at 3500 rpm for 15 min, the buffy coat was separated and mixed with 1.5 mL of RBC lysis buffer. Subsequent centrifugation and homogenization steps were followed by the addition of 1 mL of WBC lysis buffer and incubation at 60 ^∘^C. DNA was precipitated by adding chilled absolute (100%) alcohol, washed with 70% alcohol to remove contaminants, and nuclear DNA was air-dried in a fresh tube at 55 ^∘^C for 20 min [18]. The DNA from each case control was quantified and diluted to a concentration of 10 ng/µL for subsequent genotyping. TaqMan genotyping assays were employed for *ELANE:* rs17223045T/C (C__32848125_10), rs3761007G/A (C___3153452_10), and rs17216663T/C (C__60840589_20) using a 7900HT fast Real-Time PCR system (Applied Biosystems, Foster City, CA, USA). To validate our results, 70% of the samples were independently retested, ensuring concordance in all findings.

### In silico analysis

To assess the potential functional implications of *ELANE* rs17223045T/C, rs3761007A/G, and rs17216663T/C (Pro257Leu) SNVs, we employed the single nucleotide polymorphism (SNP) function prediction (FuncPred) web-based tool, available at https://snpinfo.niehs.nih.gov/snpinfo/snpfunc.html. The impact of the *ELANE* non-synonymous variant (P257L) was evaluated using PolyPhen-2, accessible at http://genetics.bwh.harvard.edu/pph2/. Additionally, we investigated the influence of these three SNVs on the expression of ELANE using the Genotype-Tissue Expression (GTEx) portal, accessible at https://gtexportal.org/home/.

### Ethical statement

Our study adhered to the principles of the Declaration of Helsinki and received approval from the Ethics, Biosecurity, and Research Committees at both the Instituto Nacional de Cardiología Ignacio Chávez and the Hospital Juárez de México, under project numbers 21-1237 and HJM 024/22-I, respectively. All patients or their relatives provided informed consent in accordance with institutional guidelines.

### Statistical analysis

Statistical analysis was conducted using SPSS version 18.0 (SPSS, Chicago, IL, USA). Continuous variables in patients with COVID-19 were compared using either the Mann–Whitney *U* test or the Student’s *t*-test. Allele and genotype frequencies of *ELANE* SNVs in patients and controls were determined through direct counting. Categorical variables were assessed using the Chi square or Fisher’s exact tests.

The possible association of *ELANE* SNVs with the susceptibility and severity of COVID-19 was assessed using logistic regression analysis under various genetic models, with adjustments for age and gender. To address multiple testing, the Bonferroni test was applied to correct *P* values (*p*C), considering a significance threshold of *≤*0.05. Associations were deemed statistically significant if the *P* value was less than or equal to 0.05. Additionally, biochemical markers were compared among different genotypes in COVID-19 patients. The data were expressed as means (± SD), comparisons were performed by ANOVA and least significant difference (LSD) as post hoc test, where *P* values < 0.05 were considered statistically significant. Linkage disequilibrium (LD) and haplotypes between *ELANE* SNVs were evaluated using Haploview (v.4.2) software.

## Results

### Clinical signs and symptoms

Table 1 presents the clinical signs, symptoms, comorbidities, and biochemical markers identified in patients with COVID-19. Notably, cough, dyspnea, myalgia, and fatigue emerged as the most prevalent signs and symptoms among the patients.

### Association analysis

The genotypic distribution of the three *ELANE* variants in the control groups adhered to Hardy–Weinberg equilibrium (HW-e) (*P* > 0.05). As shown in Table 2, allelic and genotypic frequencies of *ELANE* rs3761007G/A, rs17223045C/T, and rs17216663C/T (Pro257Leu) were examined. *ELANE* Pro257Leu exhibited a comparable distribution in both patients and controls, suggesting that this variant is not a risk factor for COVID-19. Conversely, the minor alleles of *ELANE,* rs3761007A (G vs A; odds ratio [OR] 0.38 and *P* corrected [*p*C] ═ 0.009) and rs17223045T (C vs T; OR 0.08 and *p*C ═ 0.005) were more frequent in controls than in cases, indicating statistically significant differences and a protective association against COVID-19 (Table 2). Furthermore, this association was also observed when comparing the genotype frequencies of *ELANE* rs3761007G/A (GG vs GA; OR 0.40 and *pC* ═ 0.008) and rs17223045C/T (CC vs CT; OR 0.08 and *pC* ═ 0.005) within our study group (see Table 2). Notably, no homozygous individuals carrying the minor alleles of the three *ELANE* SNVs were found among both patients and controls (Table 2). However, laboratory parameter analysis showed no association between the three exhibited *ELANE* variants and biochemical markers (Table 3).

**Table 2:** Association analysis of allele and genotype distribution of *ELANE* variants in COVID-19 patients and healthy controls

**Gene** **SNV**		**COVID-19 *n* ═ 320 (%)**	**Controls *n* ═ 288 (%)**	**OR (95% CI)**	*pC
	**rs3761007G/A**				
*ELANE*	Allele				
*Promoter region*	*G*	626 (98.1)	549 (95.3)		
	*A*	12 (1.9)	27 (4.7)	0.38 (0.19–0.77)	**0.009**
	Genotype				
	*GG*	307 (96.2)	262 (91.0)		
	*GA*	12 (3.8)	25 (8.7)	0.40 (0.20–0.83)	**0.008**
	*AA*	0 (0)	1 (0.3)		
	**rs17223045C/T**				
*ELANE exon 4*	Allele				
*Asn130Asn*	*C*	633 (99.8)	565 (98.1)		
	*T*	1 (0.2)	11 (1.9)	0.81 (0.01–0.63)	**0.005**
	Genotype				
	*CC*	316 (99.7)	277 (96.2)		
	*CT*	1 (0.3)	11 (3.8)	0.08 (0.01–0.62)	**0.005**
	*TT*	0 (0)	0 (0)		
	**rs17216663C/T**				
*ELANE exon 5*	Allele				
*Pro257Leu*	*C*	624 (99.4)	571 (99.4)		
	*T*	4 (0.6)	3 (0.6)		NS
	Genotype				
	*CC*	310 (98.7)	284 (99.0)		
	*CT*	4 (1.3)	3 (1.0)		NS
	*TT*	0 (0)	0 (0)		

Significant *P* values are reported in bold type. SNV: Single nucleotide variant; ELANE: Elastase, neutrophil-expressed; NS: No significant; COVID-19: Coronavirus disease 2019; OR: Odds ratio; *p*C: *P* corrected value; CI: Confidence interval.

**Table 3:** Association analysis of *ELANE* SNVs with biochemical markers in patients with COVID-19

***ELANE* promoter region**	**rs3761007 G/A**		
**Genotype**	**GG**	**GA**	***P* value**
Parameters	mean ± SD	mean ± SD	
Creatinine (mg/dL)	1.54 ± 2.39	2.08 ± 3.75	0.454
Ferritin (ng/µL)	849 ± 681.4	756 ± 632.7	0.687
LDH (U/dL)	412 ± 273.8	441 ± 339.4	0.721
C reactive protein (mg/dL)	66.3 ± 95.7	77.3 ± 118.2	0.723
Total bilirubin (mg/dL)	0.94 ± 1.48	1.07 ± 1.11	0.763
ALT (U/dL)	57.58 ± 56.4	55.9 ± 37.5	0.917
AST (U/dL)	58.13 ± 68.0	73.1 ± 69.7	0.455
Hemoglobin (g/dL)	13.3 ± 3.03	13.6 ± 3.16	0.737
Platelets (10^-6^/µL)	283 ± 133.2	220 ± 80.3	0.105
***ELANE Exon 5***	**rs17216663 *C/T***	**Pro257Leu**	
**Genotype**	**CC**	**CT**	***P* value**
Parameters			
Creatinine (mg/dL)	1.52 ± 2.37	0.93 ± 0.24	0.642
Ferritin (ng/µL)	847 ± 683.6	661 ± 641.3	0.639
LDH (U/dL)	414 ± 278.5	355 ± 142.1	0.672
C reactive protein (mg/dL)	67.8 ± 97.3	13.6 ± 8.35	0.267
Total bilirubin (mg/dL)	0.95 ± 1.49	1.34 ± 0.82	0.602
ALT (U/dL)	57.8 ± 56.44	48.0 ± 27.1	0.729
AST (U/dL)	58.7 ± 68.8	66.5 ± 38.5	0.821
Hemoglobin (g/dL)	13.4 ± 3.03	12.1 ± 3.07	0.394
Platelets (10^-6^/µL)	282 ± 133.10	303 ± 101.0	0.753

Data of creatinine, ferritin, LDH, C reactive protein, total bilirubin, ALT, AST, hemoglobin, and platelets are expressed as mean ± SD. LDH: Lactic acid dehydrogenase; ALT: Aminotransferase alanine; AST: Aminotransferase aspartate; ELANE: Elastase, neutrophil-expressed; SNV: Single nucleotide variant; COVID-19: Coronavirus disease 2019.

### LD and haplotypes

**Figure 1.**
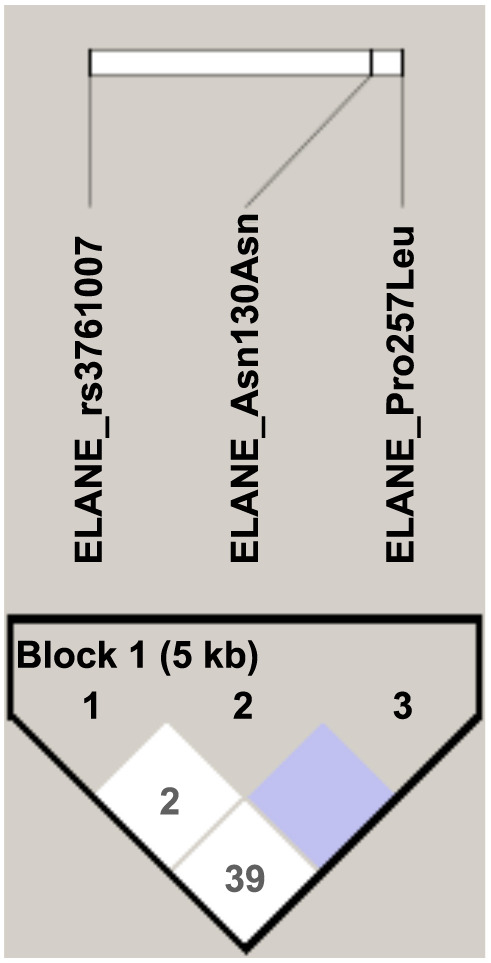
**LD between three ELANE SNVs**. None of the 3 ELANE SNVs evaluated in our case-control group showed LD. LD: Linkage disequilibrium; ELANE: Elastase, neutrophil-expressed; SNV: Single nucleotide variants.

In the co-segregation analysis between *ELANE* markers, it was determined that none of the three variants are in LD (*r*2 < 0.8) (Figure 1). On the other hand, we identified three *ELANE* haplotypes associated with COVID-19, and notably, two of these haplotypes (ACC and GTC), composed of the A allele of rs371007G/A and the T allele of rs17223045C/T, were found to be associated with protection against COVID-19 (Table 4).

**Table 4:** Association analysis of haplotype frequencies of *ELANE* SNVs in patients with COVID-19 and controls

**Haplotype**	**COVID-19 (%)**	**Controls (%)**	**OR**	**95% CI**	***P***	***pC***
GCC	606 (97.7)	530 (93.0)	3.27	1.76–6.01	1×10^-4^	0.0002
ACC	9 (1.5)	26 (4.6)	**0.31**	0.14–0.66	**0.0015**	**0.0037**
GTC	619 (99.8)	559 (98.1)	**0.08**	0.01–0.64	**0.0023**	**0.0064**

Significant *P* values are reported in bold type. *p*C: *P* corrected value after 100 000 permutations. The order is: rs3761007G/A, rs17223045C/T, and rs17216663C/T. CI: Confidence interval; OR: Odds ratio; ELANE: Elastase, neutrophil-expressed; SNV: Single nucleotide variant; COVID-19: Coronavirus disease 2019.

### *ELANE* rs17223045T/C, rs3761007A/G, and rs17216663T/C (Pro257Leu) SNV allele frequencies across populations

To compare allele frequencies between our control groups and those reported for Mexican individuals living in Los Angeles, as well as Asian, African, and Caucasian populations (as documented in the 1000 Genomes Project), we included this information in Table 5. The data revealed comparable allele frequencies between our control groups to those Mexicans in Los Angeles, and the Caucasian population. However, differences are observed in comparison to reported frequencies in Asians and Africans (Table 5).

**Table 5:** Comparison of allele frequencies between population studied and other reported groups in the 1000 genomes project

**SNV**	**Base change**	**Allele frequency in our studied population (%)**	**Allele frequency in Mexicans living in Los Angeles (%)**	**Allele frequency in Han Chinese, Beijing, China (%)**	**Allele frequency in Luhyan in Webuye, Kenya (%)**	**Allele frequency in British, England, and Scotland (%)**
**rs3761007** (promoter)	G/A	G: 95.3 A: 4.6	G: 95.3 A: 4.7	G: 74.3 A: 25.7	G: 99.0 A: 1.0	G: 92.3 A: 7.7
**rs17223045** Asn130Asn	C/T	C: 98.1 T: 1.9	C: 99.2 T: 0.8	C: 100.0 T: 0.0	C: 89.9 T: 10.1	C: 98.9 T: 1.1
**rs17216663** Pro257Leu	C/T	C: 99.4 T: 0.5	C: 98.4 T: 1.6	C: 100 T: 0	C: 100.0 T: 0.0	C: 99.5 T: 0.5

SNV: Single nucleotide variant.

### In silico analysis

Our in silico analysis using PolyPhen-2 indicated that Pro257Leu is likely benign. In contrast, Ans130Ans (rs17223045C/T) suggests that at the mRNA level, the rs17223045T allele might disrupt the binding site for the SRp40 protein, impacting splicing. Furthermore, the *ELANE* rs3761007A allele appears to disrupt the binding site for the transcription factor DR4 (Core Match Score 1, Matrix Match Score 0.71). According to the GTEx portal, *ELANE* rs17216663C/T and rs17223045C/T do not affect ELANE expression in various tissues. Meanwhile, *ELANE* rs3761007C/T appears to be an expression quantitative trait locus (eQTL) affecting its expression specifically in the thyroid (*P* ═ 0.00003).

## Discussion

The infection caused by SARS-CoV-2, leading to COVID-19, relies on the interaction between the RBD of the S protein and the human ACE2 receptor [5–7]. Additionally, various proteases play a crucial role in cleaving the S protein, including TMPRSS2, cathepsin L, and ELANE [4–8]. During viral infection, cell proteases such as TMPRSS2, utilized as a protein primer, activate the S protein by cleaving it into S1 and S2 subunits [19]. Other proteases, namely, furin, cathepsin L, and ELANE, have also been reported to cleave the SARS-CoV-2 S protein [4, 6–10]. ELANE, a serine protease primarily stored in the neutrophils’ azurophilic granules, is released into the extracellular space through degranulation or during neutrophil extracellular trap formation (NETosis). It plays a crucial role in pathogen clearance during infections and contributes to the regulation of inflammatory responses. It has been associated with various respiratory conditions, such as chronic obstructive pulmonary disease, pulmonary fibrosis, and other chronic lung diseases [20–24]. ELANE can hydrolyze the peptide bond adjacent to the polybasic amino acid sequence of the S1/S2 interface of SARS-CoV-2, suggesting its role in priming of the S1/S2 interface [10]. Studies on COVID-19 patients indicate upregulation of ELANE in nasopharyngeal swabs [11]. Moreover, elevated ELANE levels in the blood are linked to various clinical factors, including ICU admission, body temperature, lung damage, cardiovascular markers, and renal failure. ELANE is also an independent predictor of the computed tomography score of lung damage in COVID-19 [12]. Significantly elevated serum ELANE levels are seen in both ICU and non-ICU patients compared to controls, with similar patterns observed between deceased and recovered patients [13]. Another study found increased ELANE expression and NET formation in neutrophils from COVID-19 patients compared to healthy donors. Notably, phorbol myristate acetate (PMA)-elicited NET formation correlated with the severity of lung disease [25]. These findings collectively suggest a crucial role for ELANE in the pathogenesis of COVID-19.

From a genetic perspective, certain SNVs in protease genes like *TMPRSS2* have been linked to COVID-19 susceptibility or severity [26, 27]. However, SNVs in other proteases, including *ELANE*, have not been previously reported in patients with this viral infection. Our data indicate that *ELANE* rs17216663T/C (Pro257Leu) lacks association with COVID-19 susceptibility or with biochemical markers. Pro257Leu represents a non-synonymous change, and in silico analysis deems it likely benign. Furthermore, information obtained from the GTEx portal demonstrates no impact on ELANE expression in any tissue. Hence, the variant *ELANE* rs17216663T/C, not associated with COVID-19, does not appear to have a discernible biological role in protein ELANE’s structure or function, at least based on web-based tools. Conversely, both *ELANE* rs3761007G/A and Asn130Asn were found to confer protection against COVID-19. Our study represents the first to establish an association between these *ELANE* variants and COVID-19. Asn130Asn, located at exon 4, is a synonymous variant, implying that it does not result in an alteration of the amino acid sequence in the ELANE protein. Nevertheless, analysis using web-based tools suggests that at the mRNA level, the rs17223045T allele might disrupt the binding site for the SRp40 protein, a key player in splicing processes. Currently, two ELANE mRNA isoforms have been reported (https://asia.ensembl.org/Homo_sapiens/Gene/Summary?db=core;g=ENSG00000197561;r=19:851014-856247;t=ENST00000263621). Further studies investigating the functional role of the alleles of this *ELANE* SNV are imperative to determine its significance in ELANE splicing. According to the GTEx portal, this variant does not impact ELANE expression in any tissue. Additionally, the *ELANE* rs17223045C/T has been assessed in other infectious diseases, such as initial periodontitis in adolescents, and has not been associated to this pathology [28]. Concerning the *ELANE* rs3761007C/T SNV (located in its promoter), in silico analysis revealed that the T allele might disrupt the binding site for the transcription factor DR4. This suggests a potential impact on gene expression. Furthermore, ELANE rs3761007C/T appears to function as an eQTL and affects expression specifically in the thyroid. However, functional studies are necessary to ascertain the importance of this allele in ELANE expression. As far as our knowledge extends, this variant has not been evaluated in various infectious diseases, including COVID-19.

We also investigated whether the *ELANE* SNVs were associated to biochemical markers of COVID-19. However, the frequencies of the rs17216663T, rs17223045T, and rs3761007A alleles were 0.6%, 0.1%, and 1.8%, respectively. These frequencies were notably low in our patient cohort, with practically no cases carrying the heterozygous genotypes for these three variants. Consequently, upon analysis, we did not identify any association with biochemical biomarkers.

In our LD analysis, none of the three *ELANE* variants exhibited a value of *r*^2^ ≥ 0.8, indicating that these variants do not segregate together. The three haplotypes identified in our study further indicate that none of them carry two protective alleles together. This suggests that the association with protection against COVID-19 is independent for each variant.

Finally, we compared allele frequencies of the three *ELANE* variants among our controls and various populations. This revealed consistent frequencies between our controls, Mexican individuals in Los Angeles, and Caucasians. However, notable differences were observed with populations of Asian and African descent, aligning with our previous findings [8].

Our study has some limitations that should be noted: a) we did not incorporate a set of ancestry-informative markers to account for population stratification and b) the relatively small number of patients who were heterozygous and carried the minor allele for the three *ELANE* SNVs. It is crucial to emphasize that replication in other cohorts with diverse ancestry is necessary to validate their generalizability.

## Conclusion

This study represents the first to establish an association between two *ELANE* SNVs and protection against COVID-19. Nevertheless, within the parameters of our study, these variants do not function as risk or protective factors for clinical traits, comorbidities, or biochemical markers in patients with this infectious viral disease. Further investigations in diverse ethnic groups are necessary to validate and extend our findings. The outcomes of our research may contribute to identifying individuals genetically resistant to this infectious viral disease in the future.

## Data Availability

All of the data that support the findings of this study are available from the corresponding author upon reasonable request.
